# Economic efficiency analysis of different strategies to control post-weaning multi-systemic wasting syndrome and porcine circovirus type 2 subclinical infection in 3-weekly batch system farms

**DOI:** 10.1016/j.prevetmed.2012.12.006

**Published:** 2013-06-01

**Authors:** Pablo Alarcon, Jonathan Rushton, Heiko Nathues, Barbara Wieland

**Affiliations:** Royal Veterinary College, London AL9 7TA, United Kingdom

**Keywords:** Investment appraisal, Scenario analysis, Control strategies, Post-weaning multi-systemic wasting syndrome, Porcine circovirus type 2 subclinical infection

## Abstract

The study assessed the economic efficiency of different strategies for the control of post-weaning multi-systemic wasting syndrome (PMWS) and porcine circovirus type 2 subclinical infection (PCV2SI), which have a major economic impact on the pig farming industry worldwide.

The control strategies investigated consisted on the combination of up to 5 different control measures. The control measures considered were: (1) PCV2 vaccination of piglets (vac); (2) ensuring age adjusted diet for growers (diets); (3) reduction of stocking density (stock); (4) improvement of biosecurity measures (bios); and (5) total depopulation and repopulation of the farm for the elimination of other major pathogens (DPRP). A model was developed to simulate 5 years production of a pig farm with a 3-weekly batch system and with 100 sows. A PMWS/PCV2SI disease and economic model, based on PMWS severity scores, was linked to the production model in order to assess disease losses. This PMWS severity scores depends on the combination post-weaning mortality, PMWS morbidity in younger pigs and proportion of PCV2 infected pigs observed on farms.

The economic analysis investigated eleven different farm scenarios, depending on the number of risk factors present before the intervention. For each strategy, an investment appraisal assessed the extra costs and benefits of reducing a given PMWS severity score to the average score of a slightly affected farm. The net present value obtained for each strategy was then multiplied by the corresponding probability of success to obtain an expected value. A stochastic simulation was performed to account for uncertainty and variability.

For moderately affected farms PCV2 vaccination alone was the most cost-efficient strategy, but for highly affected farms it was either PCV2 vaccination alone or in combination with biosecurity measures, with the marginal profitability between ‘vac’ and ‘vac + bios’ being small. Other strategies such as ‘diets’, ‘vac + diets’ and ‘bios + diets’ were frequently identified as the second or third best strategy. The mean expected values of the best strategy for a moderately and a highly affected farm were £14,739 and £57,648 after 5 years, respectively.

This is the first study to compare economic efficiency of control strategies for PMWS and PCV2SI. The results demonstrate the economic value of PCV2 vaccination, and highlight that on highly affected farms biosecurity measures are required to achieve optimal profitability. The model developed has potential as a farm-level decision support tool for the control of this economically important syndrome.

## 1 Introduction

Porcine circovirus type 2 (PCV2), a small, non-enveloped, single stranded DNA virus, is the causative agent of several pathological conditions in the pig population worldwide. Among these conditions, post-weaning multi-systemic wasting syndrome (PMWS) is considered to be the most important (Baekbo et al., 2012). However, presence of PCV2 alone is not enough to trigger PMWS clinical signs. The necessary presence of other infectious and/or non-infectious stressor for development of clear clinical signs has been suggested in several studies (Madec et al., 2000; Alarcon et al., 2011a; Opriessnig and Halbur, 2012). As its name indicates, the main feature of PMWS is wasting or growth retardation. Multi-systemic signs, such as pneumonia, paleness and/or intermittent diarrhoea, are also frequently observed (Harding and Clark, 1997; Quintana et al., 2001). Affected pigs are normally aged between 8 and 16 weeks. At farm level, the disease increases the level of post-weaning mortality, which is often used as a reference parameter for the diagnosis of PMWS (Segales et al., 2003). Different levels of morbidity and post-weaning mortality associated with PMWS result in different disease severity levels seen on farms (Alarcon et al., 2011b). In addition to PMWS, a proportion of PCV2 infected pigs also develops a subclinical condition. These pigs, although not apparently ill, have a reduced growth rate and are believed to be more susceptible to other pathogens (Opriessnig et al., 2007; Segales, 2012). In consequence, they also contribute to the increase in post-weaning mortality. The existence of these PCV2 subclinical infected (PCV2SI) pigs became evident and widely accepted when PCV2 vaccination increased productivity in non-PMWS farms (Kurmann et al., 2011; Young et al., 2011). Both, PMWS and PCV2SI are believed to have seriously jeopardized the pig industry over the last 15 years. Their economic cost for the English pig industry was estimated around £88 million per year during the epidemic stage, and around £52.6 million during the endemic years prior the introduction of PCV2 vaccines (Alarcon et al., submitted for publication).

Known measures for the control of PMWS take into account the multi-factorial character of the disease. Several studies identified that co-infections with other pathogens, such as porcine reproductive and respiratory syndrome virus (PRRS), *Mycoplasma Hyopneumoniae*, porcine parvovirus and swine influenza virus, among others, play a major role on the development of PMWS (Krakowka et al., 2000; Pogranichniy et al., 2002; Opriessnig et al., 2004; Wellenberg et al., 2004; Dorr et al., 2007). Environmental and management factors leading to stress and increased infection pressure are also believed to trigger PMWS (Madec et al., 2000; Rose et al., 2003; Alarcon et al., 2011a). In addition, lack of essential biosecurity measures was found associated with the presence and severity of this disease at farm level (Cottrell et al., 1999; Cook et al., 2001; Woodbine et al., 2007; Alarcon et al., 2011a). The seriousness of the disease complex and its multifactorial nature led to the development of a 20 point control plan (Madec, 2001), before the development and launch of PCV2 vaccines in 2008. This plan includes a series of technical recommendations for the farrowing, post-weaning and finishing sector and is based on the improvement of hygienic conditions and within-farm biosecurity, and on the reduction of environmental stressors. However, implementing such a plan is a major investment for a farmer and only significantly reduces disease if the majority of the measures is implemented (Guilmoto and Wessel-Robert, 2000). Today, most farms use PCV2 vaccines for the control of PMWS and PCV2SI.

Despite the high efficiency of PCV2 vaccines, their cost remains an important limiting factor for the majority of farmers. Various studies showed that the vaccines are unable to eliminate the virus from the farm, and to achieve the best possible improvements, additional control measures seem to be required (Kixmoller et al., 2008; Lyoo et al., 2011; Velasova et al., submitted for publication). Further, the fact that PMWS severity varies greatly between farms indicate that different control strategies will also differ in their economic efficiency. In competitive market conditions, the need to effectively allocate the scare farm resources is essential to maintain profitability. Therefore, the aim of this study was to assess the cost-efficiency of different control strategies of single and combined control measures of PMWS and PCV2SI on farms with different PMWS severity levels.

## 2 Materials and methods

A simulation model, which represented the flow of batches on a farm operating on a 3-weekly-batch system for a period of 5 years, was developed. The cost-efficiency of different strategies, based on the combination of five different control measures, was investigated through an investment appraisal for different farm scenarios, which represented different disease severity and control or preventive measures in place. Further, a cash flow analysis was conducted to identify the maximum deficit or cash outflow of the farm, and to obtain the payback period of the investment. The control measures included were (1) PCV2 vaccination (vac), (2) age adjustment of diets of growing pigs (diet), (3) reduction of stocking density (stock), (4) improvement of biosecurity measures (bios) and (5) total depopulation/repopulation (DPRP). The last four measures were identified from the results of the farm level risk factor analysis associated with severity of PMWS (Alarcon et al., 2011a). See Table 1 for a summary of the scenario analysis approach.

### 2.1 Data sets used for this study

Data from five different studies were used to parameterise the models:

• Cross-sectional study of 147 English farms (CS-2008): this study was conducted between April 2008 and April 2009. All farms were PCV2 unvaccinated at the time of the visit. In each farm 20 blood samples (6 weaners, 6 growers, 6 finishers and 2 sows) were collected and tested for PCV2 PCR. Data on production performance, farm management, farm environment, biosecurity measures and six PMWS morbidity variables were collected with a structured questionnaire (Alarcon et al., 2011a, 2011b).
• Longitudinal study (L-2009): conducted between June 2009 and February 2010, 50 farms from the CS-2008 study were re-visited. Thirty-six farms had implemented a PCV2 vaccination programme since the first visit (Velasova et al., submitted for publication). During the second visit similar data as the CS-2008 study were collected, and PMWS severity before and after vaccination was compared.
• Longitudinal study (L-2001): this study was carried out on a commercial farm with research facilities in the United Kingdom. Between 2000 and 2001 nine batches of pigs were followed over time in an experimental study which aimed at assessing the impact of different air flow conditions on the health and growth of the pigs. Each batch was composed of 120 pigs, which were separated into 5 rooms with different environmental conditions. Throughout the experiment pig weights at animal level and feed intake at pen level were monitored for 41 days after weaning. Blood samples from 371 pigs collected at the end of the study were available for PCV2 testing through PCR. Towards the end of the experiment an outbreak of PMWS occurred and affected the last 3 batches (Wathes et al., 2004; Wieland et al., 2012).
• Farmer opinion survey conducted in 2011 (FO-2011): in this study 20 farmers were visited between June and July 2011. Data on PMWS fatality rates, veterinary and labour costs associated with PMWS, slaughterhouse carcass penalty for PMWS recovered pigs that present some degree of condemnation and cost of building proper isolated hospital pens were collected.
• UK pig industry benchmarking data: data for the years 2009 and 2010 (Bench 09 and Bench 10) were used for the baseline model (Anonymous, 2010, 2011b).

### 2.2 Farm production simulation model

**Fig. 1:**
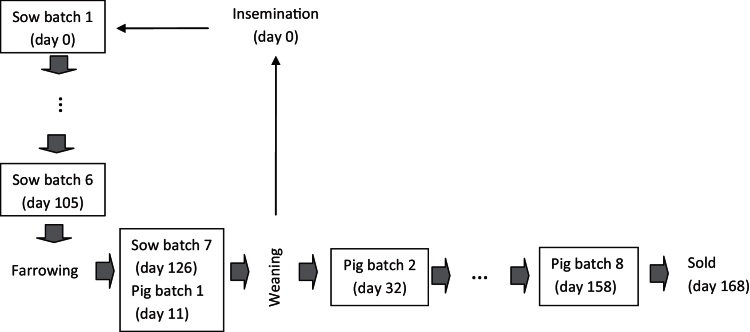
Batch production model framework of a farm operating with a 3-weekly-batch system.

A model simulating the production of batches in a 3-weekly batch system farm with 100 working sows over 5 years (1825 days) was used to assess the impact of control measures. A farm operating with a 3-weekly batch system was assumed to have at any time 7 batches of sows (sow-batch) and 8 or 9 batches of growing pigs (grower-batch) (Fig. 1). It was also assumed that pigs are weaned at 28 days of age and sent to slaughter after a further 140 days of fattening with a carcass weight of 78 kg. These parameters reflect the average production for a UK pig farm (Anonymous, 2011b). Day 0 of the model was the day of insemination of a new batch of sows. Considering that the average number of litters per sow per year was 2.25 (Bench 10), in total 91.46% of the sows will effectively deliver piglets to the farm at their corresponding time (Eq. (1)).

(1) $$\text{No. of effective sows}=\frac{100*\text{LSY}}{365/\Pi *\omega *\varpi }$$

where LSY is the average litter per sow per year (2.25), *∏* is the time of gestation of a sow (115 days), *ω* is the period of lactation of a sow (28 days) and *ϖ* is the number of days between weaning and insemination of the sow (5 days). Based on this, 8.54% of sows in each batch will fail to delivery in time, either due to returns, mortality or other causes. A farm with 100 working sows will have therefore 13.07 sows per sow-batch that will effectively deliver piglets to the farm in their corresponding time.

### 2.3 PMWS severity case definition and economic baseline model

For this study, the economic model described by Alarcon et al. (submitted for publication), which calculates the cost of PMWS and PCV2SI for farms with different PMWS severity scores, was used as a baseline. The PMWS severity was derived using the inter-correlation observed between overall post-weaning mortality, PMWS morbidity in weaners and growers age groups and the percentage of PCV2 PCR positive pigs observed on the farms included in the CS-2008 study (Alarcon et al., 2011b). The PMWS severity scale ranged between 0 and 10, and farms were classified as slightly affected (scores ≤ 4), moderately affected (scores higher than 4 and lower than 6.5) and highly affected (scores ≥ 6.5).

The baseline model accounted for pigs showing PMWS clinical signs and pigs with PCV2 subclinical infection (PCV2SI). The latter was defined as pigs with no evident clinical signs that have a slow growth rate caused by PCV2 infection and that have an increased susceptibility to other pathogens. However, the baseline mode also considered that some PCV2 infected pigs would have a normal growth rate. Therefore, the model generated six outcomes: infected pigs with clinical PMWS that die (PMWS-D); infected pigs with clinical PMWS that recover (PMWS-R); infected pigs that die due to co-infection with other pathogens (Sub-D); infected pigs with reduced growth rate that survive (Sub-S); healthy pigs, infected or not infected by PCV2, that are normally reared (H-S); and pigs, infected or not infected by PCV2, that die due to non-PCV2 related causes (nonPCV2-D). The percentage of each type of pig present in a batch at different PMWS severity scores was estimated by fitting the data on post-weaning mortality, PMWS morbidity and percentage of PCV2 PCR positive pigs from the CS-2008 study.

To assess the economic cost of disease, data on reduction of average daily gain and appetite loss of PMWS and PCV2SI were obtained from the L-2001 study by comparing data from PMWS PCV2 infected pigs, non-PMWS PCV2 infected pigs and non-PCV2 infected pigs from the batches affected by the PMWS outbreak. In addition, other costs and production parameters, such as veterinary costs, feed consumption and feed costs, water cost, straw and bedding cost, levy paid, insurance and inspection costs, labour cost, building cost, equipment cost and other fixed costs were obtained from 2010 English industry benchmarking data. An enterprise budget analysis was carried out to assess the deficit/profit of producing each type of pig (H-S, PMWS-D, PMWS-R, Sub-D, Sub-S), respectively. Subsequently, a partial budget analysis was done to assess the marginal cost and marginal profits of producing each type of diseased pig (PMWS-D, PMWS-R, Sub-D, Sub-S), respectively. The results of these economic analyses at pig level were combined with the disease model's estimates of proportion of each type of pigs at different PMWS severity scores to assess the cost of PMWS/PCV2SI and the overall profit at farm level.

### 2.4 Cost of control measures

Using the baseline economic model in combination with the farm production model, five control measures were investigated. The parameters used are summarised in Table 2 and other parameters are described by Alarcon et al. (submitted for publication).

#### 2.4.1 Improvement of biosecurity measures (bios)

Improvement of biosecurity consisted of (1) requirement of all visitors to be at least 2 days pig free (VPF), (2) improvement or creation of a hospital pens which are properly isolated (IH), and (3) closing the farm to the entrance of gilts for a period of 6 month and to the entrance of boars for the whole 5 year period (CF). The cost of implementing VPF policy on the farm was considered negligible, as it normally only requires a small change of farm management. The costs of building new isolation hospital pens were obtained through the FO-2011 study, and cost per pig per hospital pen-place was used as reference unit. The number of hospital places required on a farm was considered to be sufficient to accommodate up to 2.5% of pigs of each post-weaning grower-batch.

The third measure, CF, means that for a period of 6 month no replacement gilt is allowed to enter the farm. However, in order to achieve 20% gilts replacement rate during the 6 month closure (40% year replacement rate), the farm is assumed to buy the needed gilts, of different age groups, before the closure of the farm. Therefore, assuming that the farmer normally buys the replacement gilts at 180 days of age (7 weeks before first insemination), for this intervention at least two other batches of replacement gilts younger than 180 days (at 146 and 104 days) are bought. Thus, the extra costs and extra benefits of buying gilts at a younger age are accounted in the model. Further, due to the potential failure of some replacement gilts bought at a very young age (104 days) to develop into suitable breeding sows, for this intervention the farmer is considered to buy an additional 25% of replacement gilts of this age group. In order to be able to accommodate these young gilts, the farmer is assumed to sell weaners/growers of the corresponding age groups, and therefore, the extra costs and extra benefits of selling these weaners/growers are also accounted in the model. After the 6 month closure period, on-farm pathogens are assumed to be stabilized and only high health gilts are bought onto the farm. In addition, for the whole 5 year period no boar is bought and only semen is allowed to enter the farm. For this measure it was assumed that the farm already operates with an artificial insemination system, and therefore no new equipment costs were considered.

#### 2.4.2 Age adjusted diets (diet)

This intervention involves increasing the quality and the number of different diets for the growing pigs. For this, a 5% increase in grower feed cost was estimated.

#### 2.4.3 Reduction of stocking density (stock)

In order to reduce the stocking density of the farm, it was assumed that a farm will sell 10% of pigs just after weaning (4 weeks of age). With a partial budget analysis the extra costs and benefits of producing weaners up to 4 weeks of age were estimated and added into the model.

#### 2.4.4 Total depopulation/repopulation (DPRP)

Three different methods of DPRP were considered: (1) planned DPRP of a single farrow-to-finishing site farm (DPRP_1_), (2) planned DPRP of a multi-site farm (DPRP_2_) and (3) unplanned DPRP at day 0 (DPRP_3_). The time sequence of each DPRP strategy is shown in Table 3. In all of them, eventually all pigs are sold and the farm has to keep the breeding house empty for a minimum period of 6 weeks, and the growing/finisher houses for a minimum period of 4 weeks (Muirhead and Alexander, 2002). During the empty period no animals are allowed to remain on a site. The repopulation is done with high health sows free of any major pathogens. It was assumed that a farmer would be able to buy inseminated sows from another farm and that they would be able to transport them onto their farm 5 weeks before farrowing (80 days in gestation). In the case of DPRP_1_ and DPRP_2_, the timing of depopulation is planned so that the minimum weight of the grower sold is 30 kg.

At depopulation with DPRP_1_ and DPRP_2_, 4 and 5 batches will be sold before reaching the ideal finishing weight (unfinished batches), respectively. To calculate the profits/deficit and marginal cost/marginal benefit of selling these batches ahead of finishing, EBA and PBA were carried as described by Alarcon et al. (submitted for publication), but adjusting for the time at which each batch is finished. In the case of DPRP_3_, an unplanned depopulation, at depopulation point a farm would have 5 batches of unfinished pigs over 30 kg and 3 batches of piglets/growers less than 30 kg. These latter 3 batches were assumed to be disposed of (pigs not sold). Batches of pregnant sows would be sold. Similar as for the other DPRP methods, EBA and PBA were conducted. Other costs associated to any DPRP methods, such as the cost of cleaning and disinfection, extra labour and buying high health gilts, were inputted into the model (Table 2). For all DPRP strategies, a gap period, a period of no production, will occur. Therefore, the value of batches missed to produce was included as an intervention cost. Cost of farm maintenance during the gap period is accounted by the fact that the fix costs (labour, building, equipment and other fix costs) of the pigs that should have been produced remains unchanged (no fix cost saved).

#### 2.4.5 PCV2 vaccination (vac)

Only the piglet vaccine was considered. This vaccine is given as a single dose through intramuscular injection of 1 ml and is normally injected before weaning at about 3–4 weeks of age. The cost of a dose of PCV2 vaccine was inputted into the model for each pig weaned. In addition, the labour cost associated with the vaccination was considered.

#### 2.4.6 Time of implementation of the control measures

It was considered that PCV2 vaccination, improvement of biosecurity measures, improvement of diets and reduction of the stocking density can be implemented relatively fast and the effect will be seen in the first batch weaned in the model (new batches). Therefore the benefits were only applied to this and the following batches when sold. When any of these measures were implemented in combination with any DPRP method, the benefits were only applied to the batches of growers derived from the new batch of sows.

### 2.5 Economic analysis

#### 2.5.1 Investment appraisal (IA)

To assess the marginal costs and marginal benefits obtained from the implementation of each of the control measures, a series of investment appraisals were conducted. These investment appraisals do not take into account the effectiveness of the control strategies, which are tackled in the scenario analysis (Section 2.5.3). Instead, they consider that each control strategy is 100% effective in reducing PMWS severity from a given score to an average slightly affected severity score (2.79). Therefore, and as a first step, a basic structure for the IAs was developed to assess the marginal cost and marginal benefits of a reduction on PMWS severity. This basic structure was then modified according to the characteristic of each control measure (Table 4). For the control measure ‘stock’, two slightly different investment appraisal structures were needed because on farms with low PMWS severities, a reduction on the production of pigs, and hence a reduction of the stocking density, will not increase the number of H-S pigs, but will only reduce the number of diseased pigs. On the other hand, the reduction of stocking density in farms with high PMWS severity scores will increase the number of H-S pigs despite the reduction in the number of pigs produced. When two or more control measures were applied, the combination of the investment appraisal structure was done accordingly. When biosecurity and DPRP measures were both implemented, the cost of closing the farm for a period of 6 month was not considered, as it is no longer needed. The discount rate used to assess future cost and benefits was 3.5% (Anonymous, 2011a). For clarity purpose only, a detailed example of the investment appraisal using a deterministic approach is shown in the Appendix (Table 8 and 9). However, for this study the model was run stochastically as described in Section 2.5.4.

#### 2.5.2 Cash flow analysis

For each strategy (combination of control measures), the total cash flow for each 21 day period and for the whole 5 year period was estimated. Feasibility of each strategy was investigated by identifying the period at which profitability is obtained (payback period) and the maximum deficit or maximum investment (cash outflow) needed at one point in time. The detailed structure of the cash flow analysis is outlined in the Appendix (Table 10). The same discount rate of 3.5% was used.

#### 2.5.3 Scenario analysis (decision optimization method)

**Fig. 2:**
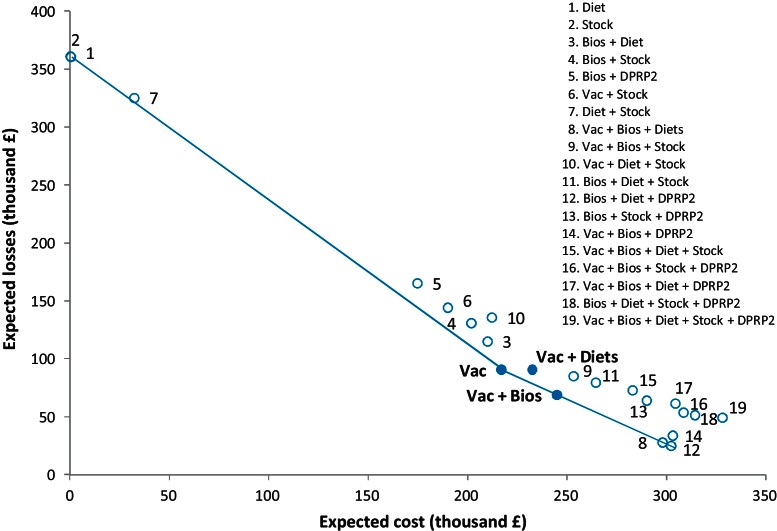
Relationship between expected losses and expected intervention costs for different strategies for farm scenario no. 4 (highly affected by PMWS and with all the risk factors present before intervention). In bold the best three strategies. The line symbolizes the loss-expenditure frontier.

The most cost-efficient strategy for the control of PMWS/PCV2SI for moderately and highly affected farms was identified with a scenario analysis based on the results of the investment appraisals. In total 11 farm scenarios were considered, each differing in the combination of at least 3 PMWS risk factors present on the farm before implementation of any control measure (Fig. 2). For each scenario, different control strategies (combination of control measures) were investigated. Strategies based on biosecurity measures alone or on DPRP without good biosecurity were not considered. The probability of a strategy to reduce the PMWS severity of a farm to an average slightly affected severity score (2.79), was derived from the odds ratios obtained from an ordinal logistic regression model described elsewhere (Alarcon et al., 2011a) using the following equation (Eq. (2)):

(2) $$P(A\cap B)=\frac{1}{1+e^{-(\alpha +A+B)}}$$

where *P*(*A *∩* B*) is the probability of a farm with risk factor A and B to be slightly affected, A is the log_*e*_ odds ratio of risk factor A, B is the log_*e*_ odds ratio of risk factor B, and *α* is the ordinal logistic regression model first intercept. The probability of success of a strategy in a given scenario was equal to the probability of being slightly affected with the risk factors remaining in the farm after the intervention. In the case of PCV2 vaccination, the probability of success was derived from the L-2009 study conducted, where 76% of moderately and highly affected farms that implemented vaccination were able to reduce their severity score to a slightly affected severity range. When PCV2 vaccination was implemented in combination with other control measures, the probability of success was estimated using the following equation (Eq. (3)):

(3) P(PCV2vac∩B)=1−((1−P(PCV2vac)×(1−P(B))

where *P*(*B*) is the probability of success of the control measure B and *P*(*PCV2vac*) is the probability of success of PCV2 vaccination alone. Table 5 shows the probabilities used in each scenario. The cost-efficiency of a strategy (*i*) was measured by the expected value (EV), which was calculated by multiplying the final net present value of the investment appraisal analysis by the corresponding probability of success (Eq. (4)).

(4) $${\text{Expected value}}_{i}=P_{i}\times {\text{net present value}}_{i}$$

For the purpose of supporting the decision-making process for each strategy a matrix with the EV, CBR, payback period, maximum deficit, maximum cash outflow at any point in time, was generated. Further, a loss-expenditure frontier was created by plotting the expected losses with the expected intervention costs of each strategy for the specific case of a multi-site farm, highly affected by PMWS and with all the risk factors present (scenario 4). Expected losses were calculated as follows (Eq. (5)):

(5) $${\text{Expetect losses}}_{i}={\text{expected losses saved}}_{p=1\;\text{and}\;c=0}-{\text{expected lossess saved}}_{i}$$

where expected losses saved_*p* = 1 and *c *= 0_ represented the total losses saved (extra revenue + cost saved) of an intervention with probability of success of 1 and with zero cost of implementation. On the other hand, the expected losses saved_*i*_ represented the losses saved (extra revenue + cost saved) of a strategy *i* multiply by the corresponding probability of success. The expected intervention cost was the sum of total extra costs and revenue forgone of an intervention, multiplied by the corresponding probability of success.

#### 2.5.4 Stochastic simulations and sensitivity analysis

To account for uncertainty and variability of the model parameters, a stochastic simulation was performed using @RISK software for Excel version 5.0 (Palisade corporation, Newfield, NY, USA). Stochastic distributions were applied to the probabilities according their 95% confidence intervals. These were obtained through bootstrapping of the multivariable ordinal logistic regression model obtained by Alarcon et al. (2011a). Bootstrapping was performed in Stata 9 (StataCorp, College station, TX) using the command prvalue (package spost9, Indiana University, USA) and the option boot to obtain the 95% percentiles of the predicted probabilities for a given combination of risk factors. Beta pert distributions were then incorporated to the probabilities by using the 95% confidence limits as minimum and maximum value, and the mean probability as the most likely value. Uncertainty on the PCV2 vaccine efficacy was introduced using the 95% confidence interval of the proportion obtained in a normal distribution (Table 6). It is important to note that all stochastic distributions of the parameters present in the baseline model were retained. Therefore the uncertainty and variability of the diagnosis protocol (PMWS severity components), production performance and others production parameters, and the disease impact variation were accounted for. The stochastic variables of the baseline model and their distributions are shown in Table 2 by Alarcon et al. (submitted for publication). The final model was run with 10,000 iterations. Sensitivity analysis was performed for cost of diets, biosecurity measures costs and costs for the reduction of stocking density and the resulting outcome was recorded. For each change the model was re-run with 1000 iterations. Mean was chosen as reference when the variable output was normally distributed. If variable output was non parametric, the median was selected.

## 3 Results

### 3.1 Results from the 3-weekly farm production model

Without any intervention, the model predicted that a farm sells a total of 87 batches in 5 years. Implementation of PCV2 vaccination, bios, diets or stock would be effective in 80 batches sold, while the other 7 batches sold would not be benefited from the interventions. In the case of DPRP_1_ and DPRP_2_, the farm would sell 9 and 10 batches not affected by the intervention and 5 and 4 unfinished batches (before reaching finishing weight), respectively. In both cases a total of 67 new batches (with intervention) would be sold, and 6 batches would be missed due to the gap period. In the case of DPRP_3_, the farm would produce 5 unfinished batches, 74 new batches, but would miss to produce 8 batches.

### 3.2 Results from the scenario tree analysis

Table 7 lists the three best profitable strategies for each scenario according to their rank on the EV. For almost all the scenarios PCV2 vaccination alone or combined with biosecurity measures was identified as the most economically efficient strategy.

On farms moderately affected by PMWS, vaccination alone was the best measure in all the scenarios. In the scenarios where biosecurity was poor, PCV2 vaccination in combination with improved biosecurity was always the second best option (scenario 3, 6–9 and 11). The average difference between ‘vac’ and ‘vac + bios’ was £8988 (difference range (£): 8850–9196) in 5 years. Besides ‘vac’ and ‘vac + bios’, ‘diets’ was also identified as an efficient strategy for these type of farms. No other strategies were identified as profitable for PMWS moderately affected farms. In four scenarios (1, 3, 6 and 9) only ‘vac’ or ‘vac + bios’ were economically efficient, with the rest of strategies having negative EV. According to the model, if the best strategy for a given scenario is implemented, the EV ranged between £13,638 and £26,947 at the end of the 5 year period (average = £14,739). The mean difference between the best and the second best option for a given scenario was £8077. The mean difference between the best and the third best option, when this third option was profitable, was £11,735.

On farms highly affected by PMWS, PCV2 vaccination was the best measure in scenarios where good biosecurity was already in place. For the other scenarios, ‘vac + bios’ was the best strategy in four of them (6, 7, 9 and 11) and ‘vac’ was the best strategy in the other three scenarios (3, 4 and 10). However the difference in EV between both strategies was frequently small. When biosecurity was initially good, ‘stock’, ‘diets’, ‘DPRP_2_’ and ‘vac + diets’ were identified as the second or third best measure. For the rest of the scenarios ‘bios + stock’, ‘bios + diets’ and ‘bios + DPRP_2_’ were identified as the third best option, always after ‘vac’ and ‘vac + bios’. Choosing the best option in each scenario would result in an EV between £53,090 and £65,975 at the end of the 5 years period (mean = £57,648). The mean difference between the best and the second best strategy for any given scenario was £5154; and the mean difference between the best and the third best strategy was £14,596.

For both, moderately and highly affected farms, no strategy including DPRP_1_ or DPRP_3_ was identified as one of the best three options. Moreover, cash flow analysis indicated that DPRP normally required the highest investment and had payback periods longer than 1 year. Of the DPRP measures, DPRP_3_ was the most expensive, as it provided the least benefits at the end of the 5 years and required the highest investment.

**Fig. 3:**
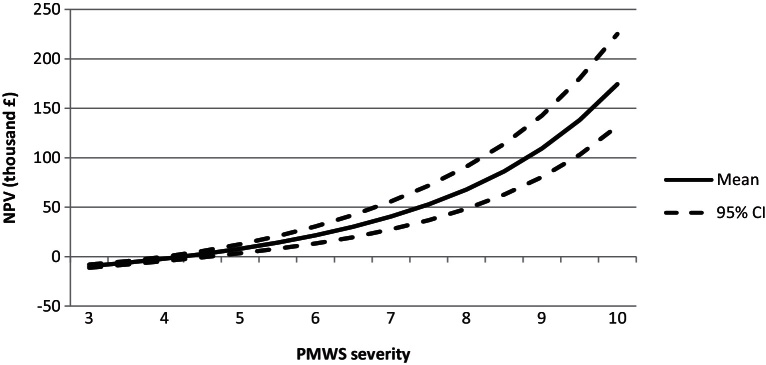
Net present value (NPV) obtained from the investment appraisal of implementing PCV2 vaccination as sole measure, and for different PMWS severities.

The losses–expenditure frontier, at which PMWS/PCV2SI can be controlled, identified ‘vac’ in the inflection point of the curve, and therefore as the best cost-efficient strategy (Fig. 2). Because of the success of the ‘vac’ strategy in the scenario analysis, this measure was further investigated. Fig. 3 shows the expected value of the investment appraisal of this strategy across the PMWS severity scale with the respective confidence intervals. Results show that ‘vac’ is only profitable on farms with PMWS severity score of 4 or higher (Fig. 3).

### 3.3 Sensitivity analysis

**Fig. 4:**
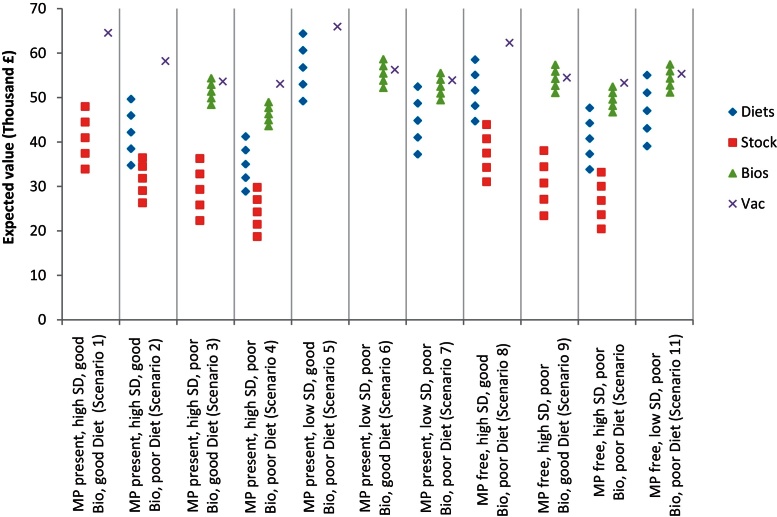
Result of the model sensitivity analysis. The graph shows the mean expected value of the best strategy containing improvement of pig diets (‘diets’), reduction in stocking density (‘stock’) or improvement of biosecurity measures (‘bios’) as control measures for each scenario and with different level of intervention of these measures (diet: 4%, 5%, 6%, 7% and 8% increase in feed cost; stock: 9%, 10%, 11%, 12% and 13% reduction in stocking density; bios: −10%, 0%, 10%, 20% and 30% change in biosecurity cost from the baseline value). The graph also shows the expected value of PCV2 vaccination alone (‘vac’), as a control strategy, without any change in its costs. The expected values are obtained for an average PMWS highly affected farm. ‘MP present/free’ means presence or freedom of major pathogens on the farm; ‘high/low SD’ means that the farm has a high or low stocking density; ‘poor/good bio’ means that the biosecurity measures considered in this study are absent or present on the farm; and ‘poor/good diet’ means that the farm do not or do adjust well enough the diets given to the age groups of the pigs.

Sensitivity analysis performed for scenarios for highly affected farms showed that a change in diet costs from 5% to 4%, 6%, 7% and 8% changed the average EV of the most successful strategy containing ‘diet’ by £3581, £3603, £7266 and £10,886 respectively. Changes in percentage of stocking density reduction from 10% to 9%, 11%, 12% and 13% changed the average EV of the most successful strategy containing ‘stock’ by £3092, £3222, £6454 and £9697 respectively. Changes of −10%, +10%, +20% and +30% in the cost of biosecurity measures changed the average EV by £1471, £1555, £3067 and £3574 respectively (Fig. 4). Changes in biosecurity cost do not alter the success of this intervention, and confirm its potential as the optimal measure for the control of PMWS and PCV2SI when combined with PCV2 vaccination. Results of the sensitivity analysis also show that in general reducing stocking density on the farm is a less profitable option than increasing the cost of diets per pig produced.

## 4 Discussion

PCV2 vaccination proved in several studies to effectively reduce disease burden on affected farms (Kristensen et al., 2011). As a consequence in the United Kingdom, as elsewhere, most of the farms have vaccinated their herds. In this study, vaccination was indeed the most efficient measures in all scenarios if the farm was moderately affected by PMWS. However, if highly affected by the disease, vaccination in combination with biosecurity measures frequently increased the expected profitability of the farm. Yet, the marginal profits that farmers will gain by implementing biosecurity measure is low, and therefore may induce farmers to adapt vaccination as the sole measure against PMWS and PCV2SI. However, good biosecurity measures might help to prevent the introduction of novel, exotic or major endemic pathogens. In the model, this is accounted for to some extend by the fact that the probability of success of ‘vac + bios’ is higher than vaccination alone. Nevertheless, situations where such diseases enter the farm could undermine the efficacy of vaccination as the sole measure. From a policy perspective, model results advice for research or implementation of policies aiming at reducing farmer's costs of biosecurity measures in order to increase the marginal expected value between both strategies. An increase in marginal value would encourage farmers to adopt strategies with biosecurity measures, such as ‘vac + bios’ instead of vaccination alone.

The efficacy of PCV2 vaccination found in this study agrees with the results of Kristensen et al. (2011). Although their meta-analysis did not consider PMWS severity scores, the average post-weaning mortality after vaccination seems to be similar to the non/slightly affected farms used in this model (3.1%). Furthermore, the estimated improvement of the batch level average daily gain of an average PMWS highly affected farm is 31.8 g, which is also in line with findings in the meta-analysis.

According to model results, in scenarios where farms had initially poor biosecurity, the implementation of biosecurity measures in combination with the vaccine, improvement of diets or reduction of stocking density was frequently observed as part of the three top strategies. Partly, this could be explained by the fact that probability of success is significantly higher when biosecurity measures are included. Probabilities of success were derived from the odds ratios identified in the ordinal logistic regression model by Alarcon et al. (2011a). As three biosecurity variables were present in the model, a simultaneous change in these three variables has a significant impact in the predicted probability. However, the fact that three measures were identified as risk factors can be considered as a reflection of the importance of biosecurity measure for the prevention of PMWS severity. Therefore the three odds ratios were considered important to estimate the predicted probabilities.

Given the high level of endemicity, it is unlikely that biosecurity measures would be able to completely prevent introduction of PCV2. Instead, biosecurity measures are important to reduce infection pressure on the farm and the entrance of other pathogens. The three biosecurity measures considered in this study were based on the risk factors identified from a large cross-sectional study in the English pig industry, and were supported by previous epidemiological studies (Cottrell et al., 1999; Cook et al., 2001; Woodbine et al., 2007). The objective of the 6 month full closure was to enable all the pigs in the farm to acquire immunity to the on-farm pathogens, and therefore reduce infection pressure. This has been proven to be effective with some pathogens such as PRRS (Scott et al., 1995). Other biosecurity measures could have been considered, as for example an all-in all-out system, effective quarantine measures, or disinfectant and other barriers at the entrance of the farm. However, these have not been identified in previous PMWS risk factor studies. Nevertheless, their potential importance as biosecurity measures suggest for further research on the model impact of their implementation.

Interestingly, the improvement of age adjusted diets or the reduction of the stocking density, in combination with other measures, was identified as the second best strategy in 3 scenarios and as part of the best third strategy in 9 scenarios for highly affected farms. However, stocking density was always more expensive than increased diets cost. A closer look indicates that in order to be in the top three, both measures needed to be in combination with at least one other measure, either already in place in the scenario or as part of the intervention. Therefore, an effective change in management and environment is needed. This reflects the multifactorial nature of the disease and the difficulties farmers have had to control it (Guilmoto and Wessel-Robert, 2000). It is also important to mention that the influence of diets on PMWS severity, although identified as a risk factor in the CS-2008 study, has not yet been validated by any other epidemiological study. Therefore, results concerning diets measures should be interpreted with care.

As with biosecurity measures, DPRP was not assumed to eliminate PCV2 from the farm completely, but to eliminate the presence of other primary pathogens that might induce the corresponding disease and thereby enhance the likelihood for pigs also to develop PMWS and PCV2SI. Only DPRP of multi-site farms (DPRP_2_) was identified as part of the top three strategies in 3 scenarios. However, as expected, this measure was identified as the least feasible option, due to its cost of implementation. Furthermore, for moderately affected farms, the risk of not having profits after implementing this intervention could be considered as high, as the EV were found negative in their low confidence intervals. Nevertheless, several externalities derived from the DPRP are not accounted in this model, and therefore the real EV could have been underestimated. In any case, the results from the model provide information of the economic advantage of multi-sites farms when DPRP is considered as an option. Further, it confirms the economic importance of carrying out planned DPRP instead of unplanned DPRP. The latter was the least profitable and feasible option (data not shown).

Several bias and limitations are present in this study. For instance, the values for the control measures, such as the increase of 5% in grower diet cost, the reduction of 10% stocking density or the 2.5% of sick places needed were chosen subjectively as the most sensible options. Sensitivity analysis showed that a change in the increase of diet cost or in the percentage of reduction of stocking density can have an important impact on the decision process in relation to these measures. However, a change in biosecurity cost did not alter significantly the model outcome, providing some flexibility for its implementation. Further, efficacies of the strategies were depended on the predicted probability obtained from the odds ratios for each risk factor from the CS-2008 study, and from the results of the L-2009 study on PCV2 vaccine efficacy. The use of bootstrapping of the ordinal logistic regression model of the PMWS risk factor study (Alarcon et al., 2011a) was a useful technique to assess the extent of uncertainty of these probabilities and to account for this uncertainty in the stochastic model. Also important was the selection of a 5 year period for the economic analysis. This seemed sensible given the nature of some of the control measures considered in this study. Depopulation and repopulation required a significant investment and to stop the production of the farm for a long time (the largest payback period obtained was 2.9 years). The DPRP strategies were combined with biosecurity measures and thus the probability of re-infection was considered minimal. Another important assumption was the price and environment stability over the 5 years. In the last 10 years, the pig industry has suffered several crises related to feed prices, pig deadweight prices, laws requiring restructuring of farms, and outbreaks of novel or exotic diseases such as foot and mouth disease, classical swine fever and PMWS (Anonymous, 2008). The possibilities of such events occurring are difficult to predict and should be considered when making important long term economic decisions. To account for possible price fluctuations over the years, algorithms based on historic data, or major cost associated to possible crises could be introduced. Nevertheless, for this study it was assumed that unexpected events that may occur on the farm during the 5 year period will affect all the farm scenarios and measures equally. It is also important to mention that some of the parameters used in this study, such as reduction on average daily weight gain and feed consumption of diseased pigs, were obtained from the L-2001 study. Although it provided data from a natural outbreak, these were derived from a single farm experience and therefore some representation bias might have occurred. Nonetheless, this model was designed as a decision support tool for farmers and veterinarians, where specific farm parameters can be introduced and therewith providing personalised results (http://www.bpex.org.uk/R-and-D/R-and-D/PMWSinPigs.aspx). Finally, limitations discussed in the models and studies used as a basis here also have influenced the outcomes (Alarcon et al., 2011a, 2011b, submitted for publication).

In order to support farmer's decision on disease control, knowing the aggregate economic impact of a disease is not sufficient and assessing the relationship between output losses and control expenditure is much more important (McInerney et al., 1992). The disease economic model captures this important concept and provides a basis for supporting farmer decisions regarding the control of PMWS and PCV2SI. In this study PCV2 vaccination was identified most frequently as the best option, which both validates the model and helps to explain the widespread use of this measure in the pig industry. However, for farms highly affected by PMWS, in half of the scenarios where farm biosecurity was poor, PCV2 vaccination in combination with good biosecurity measures was shown to be the best strategy economically. The model represents a useful decision support tool for farmers for the control of these highly economically damaging diseases, and indicates the need of further research on disease relationship with diets and stocking density.

## Conflict of interest

The authors had no conflicts of interest.

## Figures and Tables

**Table 1:** Description of the scenario analysis approach used to assess the most cost-efficient strategy against PMWS and PCV2SI.

Step one: definition of scenarios						
Scenario	Major pathogens (MP)	Stocking density (SD)	Biosecurity (bio)	Diet quality/changes (diet)	No. of different possible combinations of control measures (*i*)	PMWS severity
1	Present	High	Good	Good	15	Moderately/highly
2	Present	High	Good	Poor	28	Moderately/highly
3	Present	High	Poor	Good	18	Moderately/highly
4	Present	High	Poor	Poor	38	Moderately/highly
5	Present	Low	Good	Poor	15	Moderately/highly
6	Present	Low	Poor	Good	8	Moderately/highly
7	Present	Low	Poor	Poor	18	Moderately/highly
8	Free	High	Good	Poor	7	Moderately/highly
9	Free	High	Poor	Good	6	Moderately/highly
10	Free	High	Poor	Poor	14	Moderately/highly
11	Free	Low	Poor	Poor	6	Moderately/highly

Step two: analysis of most cost-efficient strategy
Investment appraisal → net present value_*i*_ × probability of success_*i*_ = expected value → cost–benefit ratio (in each scenario control strategies are ranked by their EV obtained)

Step three: feasibility analysis
Cash flow analysis → payback period_*i*_ → maximum investment needed_*i*_ OR maximum deficit_*i*_

**Table 2:** Parameters used for the economic model.

Parameters	Value	Reference
Cost of piglet PCV2 vaccine (£/dose)	1.41	Animeddirect.co.ik (2012)
Labour cost for the vaccination of 125 piglets	6.08	1 h × minimum UK hourly wage.
Cost of requiring visitors to be 2 days pig free (£)	0	Was considered to be negligible, as it normally requires a better organization of the farm agenda.
Cost of building a new properly isolated hospital pen (£/pig space)	131.7	Obtained from the FO11 study. Value represents average response.
Percentage of pigs that a farm should be able to accommodate in its hospital pens at maximum capacity	2.5	Selected criteria
Cost of AI insemination (£/dose)	6.06	rbst.org.uk (2012)
Number of AI doses per sow in a batch	2	Note: model accounts that some sows will not conceive after two doses and will therefore be moved to the next batch of sows, where they will receive another two doses.
Percentage of gilts purchased for replacement/year	40	Selected criteria
Numbers of boars purchased for replacement for 100 sows/year	1	Selected criteria
Cost of replacement gilts (High health) – 180 days of age (£)	200	Value obtained after consultation to breeding companies in the UK
Cost of replacement gilts with 146 days of age (£)	180	Value obtained after consultation to breeding companies in the UK
Cost of replacement gilts with 104 days of age (£)	180	Value obtained after consultation to breeding companies in the UK
Percentage of extra young gilts to be bought to account for breeding default	25	Selected criteria
Cost of replacement boars (£)	649.99	Bench 10
Revenue from breeding boar at slaughter (£)	83.3	Assume to be half the price of a sow
Breeding boar feed consumption per day (kg)	5.7	Kemp et al. (1989)
Percentage increase in grower feed cost	5	Selected criteria
Percentage reduction in stocking density	10	Selected criteria
Days in feed at which weaners are sold when reducing stocking density	0	Selected criteria
Cost of cleaning and disinfection for DPRP (£/sow)	4.29	Muirhead and Alexander (2002)
Cost of extra labour for the DPRP (£/sow)	33.57	Muirhead and Alexander (2002)
Deadweight average price per kg (DAPP)	1.39	Bench 10
Sow feed price (£/tonne)	162.87	Bench 10
Grower feed price (£/tonne)	202.55	Bench 10
Price per sow sold (£/sow)	162.58	Bench 10
Discount rate (%)	3.5	Anonymous (2011a)

**Table 3:** Time schedule of planned total depopulation and repopulation strategies (minimum weight of pigs at depopulation is 30 kg) done on single site farrow-to-finish farm (DPRP_1_) and farms with breeding and finishing pigs in separate sites (DPRP_2_); and time schedule of unplanned total depopulation/repopulation strategy (DPRP_3_, depopulation done at day 0).

	DPRP_1_	DPRP_2_	DPRP_3_
Day of insemination of last batch of old sows	1	1	–
Day of farrowing of last batch of old sows	115	115	–
Day of insemination of the first batch of new gilts (done in another farm)	140	105	1
Day of weaning of the last batch of old sows	143	143	–
Day at which all the remaining sows and gilts are sold	143	143	1
Day at depopulation of all the growing and finisher pigs	178	206	1
Period of time the breeding houses are emptied	178–220	143–185	1–42
Period of time the growing/finisher houses are emptied	178–220	192–220	1–42
Day at which the first batches of high health gilts are brought onto the farm (80 days in gestation)	220	220	80
Day at which the first batch of new gilts are farrowed	255	255	115
Day of insemination of last batch of new gilts	266	266	168
Day at which the piglets from the first batch of new gilts are weaned	308	308	143
Day at which the first batch of new gilts are sent to slaughter (full production point)	425	425	283

**Table 4:** Structure of the investment appraisal done for each control measure. In light grey are the baseline parameters, common for most of the control measures.




**Table 5:** Probabilities of success of the control measures for different farm scenarios. ‘MP present/free’ means presence or freedom of major pathogens on the farm; ‘high/low SD’ means that the farm has a high or low stocking density; ‘poor/good Bio’ means that the biosecurity measures considered in this study are absent or present on the farm; and ‘poor/good diet’ means that the farm do not or do adjust well enough the diets given to the age groups of the pigs.

	MP present, high SD, good bio, good diet (scenario 1)	MP present, high SD, good bio, poor diet (scenario 2)	MP present, high SD, poor bio, good diet (scenario 3)	MP present, high SD, poor bio, poor diet (scenario 4)	MP present, low SD, good bio, poor diet (scenario 5)	MP present, low SD, poor bio, good diet (scenario 6)	MP present, low SD, poor bio, poor diet (scenario 7)	MP free, high SD, good bio, poor diet (scenario 8)	MP free, high SD, poor bio, good diet (scenario 9)	MP free, high SD, poor bio, poor diet (scenario 10)	MP free, low SD, poor bio, poor diet (scenario 11)
Vac	0.923	0.81	0.76	0.76	0.950	0.79	0.76	0.887	0.771	0.761	0.777
Stock	0.969	0.793	0.126	0.002	–	–	–	0.943	0.383	0.071	–
Diet	–	0.681	–	0.001	0.969	–	0.126	0.902	–	0.045	0.383
Bio	–	–	–	–	–	–	–	–	–	–	–
DPRP	0.902	0.531	–	–	0.943	–	–	–	–	–	–
Bio + vac	–	–	0.923	0.81	–	0.993	0.95	–	0.976	0.887	0.986
Bio + stock	–	–	0.969	0.793	–	–	–	–	0.993	0.943	–
Bio + diet	–	–	–	0.681	–	–	0.969	–	–	0.902	0.976
Bio + DPRP	–	–	0.902	0.531	–	0.993	0.943	–	–	–	–

**Table 6:** Distributions of the probabilities of success of different control strategies obtained through bootstrapping of the multivariable logistic regression model. They correspond to the probabilities of removing the mentioned risk factors from the farm. If a risk factor is not mentioned, then it is considered to be present on the farm (i.e. ‘diets’ means that this risk factor is removed, but all the other risk factors are still present).

Combination of control measures	Mean	Range	Distribution
Diet	0.01	0.001–0.14	Beta pert
Stock	0.02	0.002–0.20	Beta pert
Bios^a^	0.21	0.01–0.88	Beta pert
DPRP^a^	0.01	0.00–0.05	Beta pert
Bios + diet	0.68	0.17–0.98	Beta pert
Bios + stock	0.79	0.18–0.99	Beta pert
Bios + DPRP	0.53	0.03–0.97	Beta pert
Diet + stock	0.13	0.03–0.56	Beta pert
DPRP + diet	0.04	0.01–0.31	Beta pert
DPRP + stock	0.07	0.01–0.48	Beta pert
Bios + diet + stock	0.97	0.72–1.00	Beta pert
Bios + diet + DPRP	0.90	0.48–1.00	Beta pert
Bios + stock + DPRP	0.94	0.35–1.00	Beta pert
Stock + diet + DPRP	0.38	0.01–0.85	Beta pert
Bios + stock + diet + DPRP	0.98	0.90–1.00	Beta pert
Vac^b^	0.76	0.58–0.93	Beta pert

a Bios and DPRP were never used alone as a control strategy, but always in combination with other control measures. DPRP was always used in combination with bios.

b Probability of success of PCV2 vaccination alone was estimated from the L-2009 study.

**Table 7:** Results of the stochastic scenario analysis. The best three economically efficient measures for each scenario and PMWS severity category are shown. All values, except ranks, are in sterling pounds. ‘MP present/free’ means presence or freedom of major pathogens on the farm; ‘high/low SD’ means that the farm has a high or low stocking density; ‘Poor/good Bio’ means that the biosecurity measures considered in this study are absent or present on the farm; and ‘poor/good diet’ means that the farm do not or do adjust well enough the diets given to the age groups of the pigs.

Scenario	PMWS severity before intervention	Ranking of control measures^a^				EV^b^ (thousand)			CBR^c^			Maximum deficit (d) or cash outflow (e) (thousand)	Payback period strategy
		Strategy	Mean rank	90% CI		Mean	90% CI		Mean	90% CI			
				Low	High		Low	High		Low	High		
MP present, high SD, good bio, good diet (scenario 1)	Moderately	Vac	1.00	1	1	16.59	9.6	24.0	1.14	1.07	1.24	1.30^e^	0.77

	Highly	Vac	1.01	1	1	64.58	44.1	86.5	1.3	1.1	1.5	1.30^e^	0.77
		DPRP2	2.74	2	5	44.06	25.4	65.0	1.2	1.1	1.3	7.01^d^	1.35
		Stock	3.05	2	6	44.50	23.6	66.7	1.2	1.1	1.5	1.02^e^	0.77

MP present, high SD, good bio, poor diet (scenario 2)	Moderately	Vac	1.00	1	1	14.96	8.6	21.8	1.14	1.07	1.24	1.30^e^	0.77
		Diet	2.00	2	2	7.95	2.6	14.4	1.09	1.03	1.17	2.82^e^	0.77

	Highly	Vac	1.08	1	2	58.22	39.3	78.8	1.3	1.1	1.5	1.30^e^	0.77
		Vac + diets	2.52	2	3	45.94	25.8	67.6	1.2	1.1	1.3	5.55^e^	0.77
		Diet	4.33	1	12	41.96	21.7	65.1	1.2	1.1	1.5	4.25^e^	0.77

MP present, high SD, poor bio, good diet (scenario 3)	Moderately	Vac	1.00	1	1	13.78	7.8	20.2	1.14	1.07	1.24	1.30^e^	0.77
		Vac + bios	2.05	2	2	4.93	−2.0	12.1	1.04	0.99	1.11	4.51^e^	0.77

	Highly	Vac	1.40	1	2	53.63	35.8	73.2	1.3	1.1	1.5	1.30^e^	0.77
		Vac + bios	1.62	1	2	52.91	32.8	74.6	1.2	1.1	1.4	4.51^e^	0.77
		Bios + DPRP2	3.73	3	6	33.62	16.2	53.9	1.1	1.1	1.2	11.17^d^	1.47

MP present, high SD, poor bio, poor diet (scenario 4)	Moderately	Vac	1.00	1	1	13.64	7.7	20.1	1.14	1.07	1.24	1.30^e^	0.77
		Vac + bios	2.24	2	4	4.44	−1.8	11.0	1.04	0.99	1.11	4.51^e^	0.77
		Diet	3.55	2	5	0.37	0.0	1.0	1.09	1.03	1.17	2.82^e^	0.77

	Highly	Vac	1.10	1	2	53.09	35.3	72.6	1.3	1.1	1.5	1.30^e^	0.77
		Vac + bios	1.98	1	2	47.71	29.2	67.9	1.2	1.1	1.4	4.51^e^	0.77
		Vac + diets	3.56	3	5	38.15	21.0	56.9	1.2	1.1	1.3	5.55^e^	0.77

MP present, low SD, good bio, poor diet (scenario 5)	Moderately	Vac	1.00	1	1	16.95	9.8	24.4	1.14	1.07	1.24	1.30^e^	0.77
		Diet	2.00	2	2	11.49	4.3	19.0	1.09	1.03	1.17	2.82^e^	0.77

	Highly	Vac	1.11	1	2	65.97	45.1	88.1	1.3	1.1	1.5	1.30^e^	0.77
		Diet	1.94	1	2	60.62	39.8	83.0	1.2	1.1	1.5	4.25^e^	0.77
		Vac + diets	3.47	3	5	49.59	28.2	72.2	1.2	1.1	1.3	5.55^e^	0.77

MP present, low SD, poor bio, good diet (scenario 6)	Moderately	Vac	1.00	1	1	14.46	8.2	21.1	1.14	1.07	1.24	1.30^e^	0.77
		Vac + bios	2.00	2	2	5.32	−2.2	13.0	1.04	0.99	1.11	4.51^e^	0.77

	Highly	Vac + bios	1.49	1	2	57.11	35.7	79.7	1.2	1.1	1.4	4.51^e^	0.77
		Vac	1.54	1	2	56.27	37.8	76.2	1.3	1.1	1.5	1.30^e^	0.77
		Bios + DPRP2	2.98	3	3	38.81	19.5	60.6	1.1	1.1	1.2	11.17^d^	1.47

MP present, low SD, poor bio, poor diet (scenario 7)	Moderately	Vac	1.00	1	1	13.85	7.9	20.3	1.14	1.07	1.24	1.30^e^	0.77
		Vac + bios	2.23	2	3	5.03	−2.1	12.4	1.04	0.99	1.11	4.51^e^	0.77
		Diet	3.12	2	5	2.20	0.3	5.2	1.09	1.03	1.17	2.82^e^	0.77

	Highly	Vac + bios	1.61	1	2	54.06	33.4	76.0	1.2	1.1	1.4	4.51^e^	0.77
		Vac	1.68	1	3	53.91	36.1	73.5	1.3	1.1	1.5	1.30^e^	0.77
		Bios + diet	2.79	2	3	48.68	28.0	70.8	1.2	1.1	1.4	7.46^e^	0.77

MP free, high SD, good bio, poor diet (scenario 8)	Moderately	Vac	1.00	1	1	16.01	9.2	23.3	1.14	1.07	1.24	1.30^e^	0.77
		Diet	2.00	2	2	10.45	3.9	17.7	1.09	1.03	1.17	2.82^e^	0.77

	Highly	Vac	1.15	1	2	62.34	42.2	84.0	1.3	1.1	1.5	1.30^e^	0.77
		Diet	2.01	1	3	55.09	34.9	77.9	1.2	1.1	1.5	4.25^e^	0.77
		Vac + diets	2.91	2	3	48.51	27.4	70.8	1.2	1.1	1.3	5.55^e^	0.77

MP free, high SD, poor Bio, good diet (scenario 9)	Moderately	Vac	1.00	1	1	14.00	7.9	20.5	1.14	1.07	1.24	1.30^e^	0.77
		Vac + bios	2.01	2	2	5.20	−2.1	12.8	1.04	0.99	1.11	4.51^e^	0.77

	Highly	Vac + bios	1.44	1	2	55.87	34.8	78.2	1.2	1.1	1.4	4.51^e^	0.77
		Vac	1.56	1	2	54.49	36.5	74.2	1.3	1.1	1.5	1.30^e^	0.77
		Bios + stock	3.08	3	4	34.44	12.8	57.3	1.2	1.0	1.4	4.23^e^	0.77

MP free, high SD, poor Bio, poor diet (scenario 10)	Moderately	Vac	1.00	1	1	13.70	7.7	20.1	1.14	1.07	1.24	1.30^e^	0.77
		Vac + bios	2.22	2	4	4.75	−2.0	11.7	1.04	0.99	1.11	4.51^e^	0.77
		Diet	3.40	2	5	0.97	0.1	2.5	1.09	1.03	1.17	2.82^e^	0.77

	Highly	Vac	1.38	1	3	53.31	35.6	72.8	1.3	1.1	1.5	1.30^e^	0.77
		Vac + bios	1.84	1	3	51.08	31.4	72.4	1.2	1.1	1.4	4.51^e^	0.77
		Bios + diet	3.03	1	4	44.24	24.9	66.2	1.2	1.1	1.4	7.46^e^	0.77

MP free, low Sd, poor bio, poor diet (scenario 11)	Moderately	Vac	1.00	1	1	14.22	8.1	20.8	1.14	1.07	1.24	1.30^e^	0.77
		Vac + bios	2.52	2	3	5.21	−2.1	12.7	1.04	0.99	1.11	4.51^e^	0.77
		Diet	2.58	2	4	4.93	1.1	10.2	1.09	1.03	1.17	2.82^e^	0.77

	Highly	Vac + bios	1.52	1	2	55.95	34.9	78.2	1.2	1.1	1.4	4.51^e^	0.77
		Vac	1.72	1	3	55.35	37.1	75.1	1.3	1.1	1.5	1.30^e^	0.77
		Bios + diet	2.78	2	3	51.07	29.7	73.8	1.2	1.1	1.4	7.46^e^	0.77

a Strategies are ranked according to their expected value in each stochastic iteration.

b EV means expected value, which is equal to the net present value obtained in the investment appraisal multiply by the corresponding probability of success.

c CBR means cost–benefit ratio, which equals the expected revenue divided by the expected cost obtained from the investment appraisals.

d Maximum deficit: maximum negative income obtained at one point in time.

e The maximum cash outflow represents the largest amount of money that a farmer will need to pay at one point in time. This was only reported when the farmer never incurred into a deficit by implementing the control strategy. It was calculated as the sum of all the costs of a control strategy for the first seven batches. Seven batches were considered as they represent the sow batch cycle in a 3-weekly-batch system farm. The corresponding payback period is therefore the time until the 7th batch post-intervention is sent for slaughter.
