# Supplementary material for: Economic efficiency analysis of different strategies to control post-weaning multi-systemic wasting syndrome and porcine circovirus type 2 subclinical infection in 3-weekly batch system farms
Source: Prev Vet Med. 2013 Jun 1;110(2):103–18. doi: 10.1016/j.prevetmed.2012.12.006 (PMC3652493; doi:10.1016/j.prevetmed.2012.12.006)
Supplement: Supplementary file 1 [file mmc1.docx]

**Table 10.** Structure for the cash-flow analysis for each control measure. In light grey are the baseline parameters, common for most of the control measures.

|  | **Costs** | **Revenue** |
| --- | --- | --- |
| Basic structure (severity reduction without any control measure) | - Costs of feed, water, electricity, veterinary, straw & bedding, ILL, and transport cost or carcass disposal cost of H-S, PMWS-D, PMWS-S, Sub-D and Sub-S from old batches sold - Costs of feed, water, electricity, veterinary, straw & bedding, ILL, and transport cost/carcass disposal cost of H-S, PMWS-D, PMWS-S, Sub-D and Sub-S from new batches sold - Costs on Labour, building, equipment and other fix cost from old batches - Costs on Labour, building, equipment and other fix cost from new batches | - Revenue from H-S, PMWS-S and Sub-S from old batches sold - Revenue from H-S, PMWS-S and Sub-S from new batches sold - Sow depreciation of old batches - Sow depreciation of sows from new batches |
| PCV2 vaccination | - Costs of feed, water, electricity, veterinary, straw & bedding, ILL, and transport cost or carcass disposal cost of H-S, PMWS-D, PMWS-S, Sub-D and Sub-S from old batches sold - Costs of feed, water, electricity, veterinary, straw & bedding, ILL, and transport cost/carcass disposal cost of H-S, PMWS-D, PMWS-S, Sub-D and Sub-S from new batches sold - Costs on Labour, building, equipment and other fix cost from old batches - Costs on Labour, building, equipment and other fix cost from new batches - Cost of PCV2 vaccination | - Revenue from H-S, PMWS-S and Sub-S from old batches sold - Revenue from H-S, PMWS-S and Sub-S from new batches sold - Sow depreciation of old batches - Sow depreciation of sows from new batches |
| Biosecurity measures | - Costs of feed, water, electricity, veterinary, straw & bedding, ILL, and transport cost or carcass disposal cost of H-S, PMWS-D, PMWS-S, Sub-D and Sub-S from old batches sold - Costs of feed, water, electricity, veterinary, straw & bedding, ILL, and transport cost/carcass disposal cost of H-S, PMWS-D, PMWS-S, Sub-D and Sub-S from new batches sold - Costs on Labour, building, equipment and other fix cost from old batches - Costs on Labour, building, equipment and other fix cost from new batches - Cost of requiring visitors to be pig free - Cost of new sick/hospital pen - Cost of purchasing AI - Correction for cost saved from not buying boars - Cost of purchasing extra gilts bought to account for breeding default - Correction for the cost saved on replacement gilts bought at younger age - Cost of feed, water, electricity, veterinary, straw & bedding cost of young gilts bought until reaching 180 days of age - Correction for feed, Vet&Med, elect., water and bedding cost saved from weaners that were sold to accommodate extra gilts. | - Revenue from H-S, PMWS-S and Sub-S from old batches sold - Revenue from H-S, PMWS-S and Sub-S from new batches sold - Sow depreciation of old batches - Sow depreciation of sows from new batches - Correction: revenue forgone from the weaners that were sold to accommodate extra gilts brought onto the farm. - Correction for revenue forgone from selling breeding boar to slaughter - Revenue from gilts with breeding default that are sent to slaughter |

**Table 8.** continue

|  | **Costs** | **Revenue** |
| --- | --- | --- |
| Improvement of diets | - Costs of feed, water, electricity, veterinary, straw & bedding, ILL, and transport cost or carcass disposal cost of H-S, PMWS-D, PMWS-S, Sub-D and Sub-S from old batches sold - Costs of feed, water, electricity, veterinary, straw & bedding, ILL, and transport cost/carcass disposal cost of H-S, PMWS-D, PMWS-S, Sub-D and Sub-S from new batches sold - Costs on Labour, building, equipment and other fix cost from old batches - Costs on Labour, building, equipment and other fix cost from new batches - Extra cost of feed of H-S, PMWS-D, PMWS-S, Sub-D and Sub-S from new batches | - Revenue from H-S, PMWS-S and Sub-S from old batches sold - Revenue from H-S, PMWS-S and Sub-S from new batches sold - Sow depreciation of old batches - Sow depreciation of sows from new batches |
| Reduction of stocking density | - Costs of feed, water, electricity, veterinary, straw & bedding, ILL, and transport cost or carcass disposal cost of H-S, PMWS-D, PMWS-S, Sub-D and Sub-S from old batches sold - Costs of feed, water, electricity, veterinary, straw & bedding, ILL, and transport cost/carcass disposal cost of H-S, PMWS-D, PMWS-S, Sub-D and Sub-S from new batches sold - Costs on Labour, building, equipment and other fix cost from old batches - Costs on Labour, building, equipment and other fix cost from new batches (including from weaners sold) - Costs of feed, water, electricity, veterinary, straw & bedding, ILL, and transport cost of weaners sold from new batches | - Revenue from H-S, PMWS-S and Sub-S from old batches sold - Revenue from H-S, PMWS-S and Sub-S from new batches sold - Sow depreciation of old batches - Sow depreciation of sows from new batches - Revenue from selling weaners |
| DPRP_1_ and DPRP_2_ | - Costs of feed, water, electricity, veterinary, straw & bedding, ILL, and transport cost or carcass disposal cost of H-S, PMWS-D, PMWS-S, Sub-D and Sub-S from old batches sold - Costs of feed, water, electricity, veterinary, straw & bedding, ILL, and transport cost/carcass disposal cost of H-S, PMWS-D, PMWS-S, Sub-D and Sub-S from new batches sold - Costs on Labour, building, equipment and other fix cost from old batches - Costs on Labour, building, equipment and other fix cost from new batches - Cost of cleaning & disinfection and extra labour during the empty period. - Cost of buying high health free gilts - Costs of feed, water, electricity, veterinary, straw & bedding, ILL, and transport cost or carcass disposal cost of H-S, PMWS-D, PMWS-S, Sub-D and Sub-S from unfinished batches sold - Costs on Labour, building, equipment and other fix cost from unfinished batches - Cost of Labour, building, equipment and other fix cost from batches non produced during the gap period | - Revenue from H-S, PMWS-S and Sub-S from old batches sold - Revenue from H-S, PMWS-S and Sub-S from new batches sold - Sow depreciation of sows from new batches - Revenue from H-S, PMWS-S and Sub-S from unfinished batch sold - Revenue from selling sows |

**Table 8.** continue

|  | **Costs** | **Revenue** |
| --- | --- | --- |
| DPRP_3_ | - Costs of feed, water, electricity, veterinary, straw & bedding, ILL, and transport cost or carcass disposal cost of H-S, PMWS-D, PMWS-S, Sub-D and Sub-S from old batches sold - Costs of feed, water, electricity, veterinary, straw & bedding, ILL, and transport cost/carcass disposal cost of H-S, PMWS-D, PMWS-S, Sub-D and Sub-S from new batches sold - Costs on Labour, building, equipment and other fix cost from old batches - Costs on Labour, building, equipment and other fix cost from new batches - Cost of cleaning & disinfection and extra labour during the empty period. - Cost of buying high health free gilts - Costs of feed, water, electricity, veterinary, straw & bedding, ILL, and transport cost or carcass disposal cost of H-S, PMWS-D, PMWS-S, Sub-D and Sub-S from unfinished batches sold and unfinished batches not sold - Costs on Labour, building, equipment and other fix cost from unfinished batches sold and unfinished batches not sold. - Cost of Labour, building, equipment and other fix cost from batches non produced during the gap period | - Revenue from H-S, PMWS-S and Sub-S from old batches sold - Revenue from H-S, PMWS-S and Sub-S from new batches sold - Sow depreciation of sows from new batches - Revenue from H-S, PMWS-S and Sub-S from unfinished batch sold - Revenue from selling sows |
