# Supplementary material for: Economic efficiency analysis of different strategies to control post-weaning multi-systemic wasting syndrome and porcine circovirus type 2 subclinical infection in 3-weekly batch system farms
Source: Prev Vet Med. 2013 Jun 1;110(2):103–18. doi: 10.1016/j.prevetmed.2012.12.006 (PMC3652493; doi:10.1016/j.prevetmed.2012.12.006)
Supplement: Supplementary file 2 [file mmc2.docx]

| Number of batches  **Table 8. Example of Partial Budget Analysis for the PCV2 vaccination model (reduction of severity from 7.52 to 2.92) – Deterministic output** | | | | | | | | | Types of pigs / batch | | | | | | | | | | |
| --- | --- | --- | --- | --- | --- | --- | --- | --- | --- | --- | --- | --- | --- | --- | --- | --- | --- | --- | --- |
|  | Y1 | Y2 | Y3 | Y4 | | Y5 | Total | |  | | | H-S | | PMWS-D | | PMWS-R | | Sub-D | Sub-S |
| If no intervention:  Old  If intervention:  Old  new | 17  7  10 | 18  0  18 | 17  0  17 | 19  0  19 | | 16  0  17 | 87  7  80 | | Old:  New:  Difference: | | | 78  109.5  31.5 | | 6.2  0  -6.2 | | 1.5  0  -1.5 | | 7.1  0  -7.1 | 30.7  14  -16.7 |
| **Partial Budget Analysis (000s)** | | | | | | | | | | | | | | | | | Reference | | |
| **Extra cost**  PCV2 vaccination  Extra feed on new H-S  Extra Vet&Med on new H-S  Extra elect on new H-S  Extra water on new H-S  Extra bedding on new H-S  Extra transport on new H-S  Extra ILL on new H-S  **Revenue forgone**  Revenue missed on PMWS-S  Revenue missed on Sub-S  **Cost saved**  Feed saved on PMWS-D  Feed saved on PMWS-S  Feed saved on Sub-D  Feed saved on Sub-S  Vet&Med on PMWS-S  Vet&Med on PMWS-R  Vet&Med on Sub-D  Vet&Med on Sub-S  Elect. Saved on PMWS-D  Elect. Saved on PMWS-S  Elect. Saved on Sub-D  Elect. Saved on Sub-S  Water saved on PMWS-D  Water saved on PMWS-R  Water saved on Sub-D  Water saved on Sub-S  Bedding saved on PMWS-D  Bedding saved on PMWS-R  Bedding saved on Sub-D  Bedding saved on Sub-S  Carcass disposal saved on PMWS-D  Carcass disposal saved on Sub-D  Transport saved on PMWS-R  Transport saved on Sub-S  ILL saved on PMWS-R  ILL saved on Sub-S  **New revenue**  Carcass sold of new H-S  **Total/(1+r)^y^** | | | | | Y1  2.97  14.41  0.59  0.67  0.19  0.14  0.74  0.91  1.50  18.09  0.95  0.75  1.36  8.16  0.39  0.12  0.06  0.37  0.05  0.04  0.07  0.42  0.01  0.01  0.02  0.12  0.01  0.01  0.01  0.09  0.37  0.43  0.04  0.39  0.04  0.48  34.16  8.43 | | | Y2  3.34  25.95  1.06  1.20  0.34  0.24  1.33  1.64  2.71  32.54  1.71  1.36  2.44  14.69  0.70  0.21  0.11  0.67  0.09  0.07  0.13  0.76  0.03  0.02  0.04  0.21  0.02  0.01  0.03  0.15  0.67  0.77  0.06  0.70  0.08  0.87  61.48  16.54 | | Y3  3.16  24.51  1.01  1.14  0.32  0.23  1.25  1.55  2.56  30.73  1.61  1.28  2.30  13.87  0.66  0.20  0.10  0.63  0.09  0.07  0.12  0.72  0.03  0.02  0.03  0.20  0.02  0.01  0.02  0.15  0.63  0.72  0.06  0.66  0.07  0.82  58.07  15.09 | Y4  3.53  27.39  1.11  1.27  0.36  0.26  1.40  1.74  2.86  34.35  1.80  1.43  2.58  15.51  0.74  0.22  0.12  0.71  0.10  0.08  0.13  0.80  0.03  0.02  0.04  0.23  0.02  0.02  0.03  0.16  0.71  0.81  0.07  0.74  0.08  0.92  64.90  16.30 | | Y5  3.16  23.07  0.94  1.07  0.30  0.2161.18  1.46  2.41  28.93  1.52  1.21  2.17  13.06  0.62  0.19  0.10  0.59  0.08  0.06  0.11  0.67  0.02  0.02  0.03  0.19  0.02  0.01  0.02  0.14  0.60  0.68  0.06  0.62  0.07  0.77  54.65  13.10 | | Total  16.15  115.33  4.71  5.34  1.51  1.08  5.90  7.31  12.04  14.46  7.59  6.03  10.85  72.67  3.10  0.95  0.49  2.97  0.42  0.32  0.56  3.37  0.12  0.09  0.16  0.95  0.09  0.06  0.11  0.68  2.98  3.41  0.28  3.12  0.35  3.87  273.25  69.47 | | =no. weaned in new batches * (cost of PCV2 dose + labour cost per pig)  =no. extra H-S*feed cost/H-S^1^  =no. extra H-S*Vet&Med/H-S^1^  =no. extra H-S*elect./H-S^1^  =no. extra H-S*water/H-S^1^  =no. extra H-S*bedding/H-S^1^  =no. extra H-S*transport/H-S^1^  =no. extra H-S*ILL/H-S^1^  =no. PMWS-R missed*PMWS-R carcass value^1^  =no. Sub-S missed*Sub-S carcass value^1^  =no. PMWS-D missed*feed/PMWS-D^1^  =no. PMWS-R missed*feed/PMWS-R^1^  =no. Sub-D missed*feed/Sub-D^1^  =no. Sub-S missed*feed/Sub-S^1^  =no. PMWS-D missed*Vet&Med/PMWS-D^1^  =no. PMWS-R missed*Vet&Med/PMWS-R^1^  =no. Sub-D missed*Vet&Med/Sub-D^1^  =no. Sub-S missed*Vet&Med/Sub-S^1^  =no. PMWS-D missed*Elect/PMWS-D^1^  =no. PMWS-R missed*Elect/PMWS-R^1^  =no. Sub-D missed*Elect/Sub-D^1^  =no. Sub-S missed*Elect/Sub-S^1^  =no. PMWS-D missed*Water/PMWS-D^1^  =no. PMWS-R missed*Water/PMWS-R^1^  =no. Sub-D missed*Water/Sub-D^1^  =no. Sub-S missed*Water/Sub-S^1^  =no. PMWS-D missed*Water/PMWS-D^1^  =no. PMWS-R missed*Water/PMWS-R^1^  =no. Sub-D missed*Water/Sub-D^1^  =no. Sub-S missed*Water/Sub-S^1^  =no. PMWS-D missed*Carc dispossal/PMWS-D^1^  =no. PMWS-R missed*Carc dispossal/PMWS-R^1^  =no. Sub-D missed*transport/Sub-D^1^  =no. Sub-S missed*transport/Sub-S^1^  =no. PMWS-R missed*ILL/PMWS-R^1^  =no. Sub-S missed*ILL/Sub-S^1^  =no. extra H-S*carcass value/H-S | | |

^1^See table 4 in Alarcon et al. (2012)
