# Supplementary material for: Economic efficiency analysis of different strategies to control post-weaning multi-systemic wasting syndrome and porcine circovirus type 2 subclinical infection in 3-weekly batch system farms
Source: Prev Vet Med. 2013 Jun 1;110(2):103–18. doi: 10.1016/j.prevetmed.2012.12.006 (PMC3652493; doi:10.1016/j.prevetmed.2012.12.006)
Supplement: Supplementary file 3 [file mmc3.docx]

| Number of batches/animals/weaners  **Table 9. Example of Partial Budget Analysis for the ‘Vac+Bios’ model (reduction of severity from 7.52 to 2.92) – Deterministic output** | | | | | | | | | | | Types of pigs / batch | | | | | | | |
| --- | --- | --- | --- | --- | --- | --- | --- | --- | --- | --- | --- | --- | --- | --- | --- | --- | --- | --- |
|  | Y1 | Y2 | | Y3 | | Y4 | | Y5 | | Total |  | | H-S | | PMWS-D | PMWS-R | Sub-D | Sub-S |
| If no intervention:  Old    If intervention:  Old  New  No. of sow batches inseminated:  No. of gilts bought:  146 days of age  104 days of age  No. of weaners sold to accommodate for gilts: | 17  7  10  16  6  4  14 | 18  0  18  18 | | 17  0  17  17 | | 19  0  19  19 | | 16  0  16  17 | | 87  7  80  88 | Before:  After:  Difference: | | 78  109.5  31.5 | | 6.2  0  -6.2 | 1.5  0  -1.5 | 7.1  0  -7.1 | 30.7  14  -16.7 |
| **Partial Budget Analysis (000s)** | | | | | | | | | | | | | | Reference | | | | |
| **Extra cost**  PCV2 vaccination  Cost of being pig free  Cost of creating sick isolated pens  Cost of purchasing AI  Buying 25% extra gilts, at young age (<146days),  to account for breeding default  Cost of feed, Vet&Med, elect., water and  bedding of young gilts (<180 days) bought,  and that will use for breeding, until reaching  180 days of age.  Cost of feed, Vet&Med, elect., water and  bedding of young gilts (<180 days) bought  and that will be slaughter at 168 days due to  breeding default  Extra feed, Vet&Med, elect., water, bedding, ILL  and transport on new H-S  **Revenue forgone**  Revenue missed on PMWS-S and Sub-S  Revenue missed from breeding boars  Revenue forgone from the weaners sold to accommodate new gilts  **Cost saved**  Saved on boar replacement  Feed, Vet&Med, elec., water , bedding, ILL and  transport saved on breeding board  Feed, Vet&Med, elec., water , bedding, ILL and  transport saved on weaners sold  Saved of buying gilts at younger age  Feed, Vet&Med, elec., water, bedding, ILL and  transport/carcass disposal saved on PMWS-D,  PMWS-R, Sub-D and Sub-S missed  **New revenue**  Carcass sold of new H-S  Revenue from extra gilts with breeding default  that are sent to slaughter  **Total/(1+r)^y^** | | | Y1  -2.97  0  -2.94  -2.92  -0.18  -0.12  -0.02  -17.65  -19.58  0  -0.59  0.65  0.43  0.14  0.25  14.77  34.16  0.27  3.81 | | Y2  -3.34  0  0  -3.09  0  0  0  -31.78  -32.25  -0.16  0  0.65  0.43  0  0  26.60  61.48  0  14.51 | | Y3  -3.16  0  0  -2.92  0  0  0  -30.00  -33.29  -0.16  0  0.65  0.43  0  0  25.12  58.07  0  13.29 | | Y4  -3.41  0  0  -3.26  0  0  0  -33.53  -37.21  -0.16  0  0.65  0.43  0  0  28.08  64.90  0  14.26 | | Y5  -3.16  0  0  -2.92  0  0  0  -28.24  -31.33  -0.16  0  0.65  0.43  0  0  23.64  54.65  0  11.42 | Total  -16.15  0  -2.94  -15.09  -0.18  -0.12  -0.02  -141.19  -156.67  -0.65  -0.59  3.25  2.15  0.14  0.25  118.22  273.25  0.27  56.67 | | =no. weaned in new batches * (cost of PCV2 dose + labour cost per pig)  =assumed to be zero  =cost/pig place * (no. pigs weaned in 7 bacthes*2.5%)  =price of AI dose * no.sow bacthes insem. * no sows/batch  =no. of gilts bought <146 days * 0.025 * corresponding price of gilt  =extrapolation of each variable cost per day for a H-S pig to the no. of extra days the young gilts remain on the farm * no of young gilts bought that will be use for breeding  =extrapolation of each variable cost per day for a H-S pig to the no. of extra days the young gilts remain on the farm * no of extra gilts bought that will be sent to slaughter at 168 days  =similar calculations as Table 9  =similar calculations as in Table 9  =no. breed. boar sold per year *price of breed. at slaughter  = (price/liveweight kg. for weaner pigs) * liveweight of weaners at 146, 104 and 84 days of age * no. of weaners sold in each age group  =no. breeding boars bough * price of breeding boar  =extrapolation of variable cost per day for a H-S pig * 365. In the case of feed it was assume that a boar eats 5.7kg. per day, and sow feed price was used.  = from the adaptation of the enterprise budget analysis of an H-S pig (see Table 3 in Alarcon et al., 2012) to the corresponding age of weaners.  =differencial between prices of gilts at 180 days and price of gilts <180days * (no. gilts <180 days bought – no. extra gilts<180 days bought due to breeding default)  =similar calculations as in Table 9  =similar calculations as in Table 9  =similar calculations as in Table 9  =liveweight of gilts at 168 days * deadweight price/kg.*no. of gilts bought with breeding default sent to slaughter | | | | |
